# Abundance of commercially important reef fish indicates different levels of over-exploitation across shelves of the U.S. Virgin Islands

**DOI:** 10.1371/journal.pone.0180063

**Published:** 2017-07-13

**Authors:** Elizabeth Kadison, Marilyn Brandt, Richard Nemeth, Justin Martens, Jeremiah Blondeau, Tyler Smith

**Affiliations:** 1 Center for Marine and Environmental Studies, University of the Virgin Islands, St. Thomas, USVI, United States of America; 2 U.S. Fish and Wildlife Service, Alpine, AZ, United States of America; 3 NOAA Southwest Fisheries Science Center, 75 Virginia Beach Dr., Miami, FL, United States of America; Department of Agriculture and Water Resources, AUSTRALIA

## Abstract

The United States Virgin Islands are comprised of two separate insular platforms separated by the deep water Anegada Passage. Although managed by the same regulations, as one fishery, several physical and spatial differences exist between the two northern shelf islands, St. Thomas and St. John, and isolated St. Croix. Based on two long-term fisheries independent datasets, collected by the U.S. Virgin Islands Territorial Coral Reef Monitoring Program and the National Oceanographic and Atmospheric Administration Center for Coastal Monitoring and Assessment, there were significant differences between the northern USVI and St. Croix in both the occurrence and size of several species of large and commercially important reef fishes. These fishes are primarily apex piscivores and generally the first species over-exploited in small-scale fisheries. The disparities between the fish communities on the two island shelves cannot be explained solely by differences in habitat (coral cover, rugosity) or fisheries management, such as relative amount of marine protected area in local waters. They are instead probably caused by a combination of several other interrelated factors including water depth, fishing methodology, fishable area, and the presence or absence of viable fish spawning areas. This study considers those aspects, and illustrates the need for management of island artisanal fisheries that is tailored to the physical and spatial constraints imposed by insular platforms.

## Introduction

Across the wider Caribbean, temporal stock declines and changes in reef fish communities have been well documented [1–6]. Degradation of benthic habitats has contributed to these changes, however there is clear evidence that overexploitation led by the modernization of small-scale artisanal fisheries has been the major cause of fisheries resource decline in the region. In particular, large, highly-valued predatory species such as groupers and snappers have shown the most notable declines [7–10], a trend also documented in artisanal fisheries in islands of the Pacific [11–12]. Groupers and snappers are particularly vulnerable to over-exploitation due to their slow growth rate, late maturity, sporadic reproduction and recruitment, and vulnerability during spawning aggregations [13–16]).

**Fig 1:**
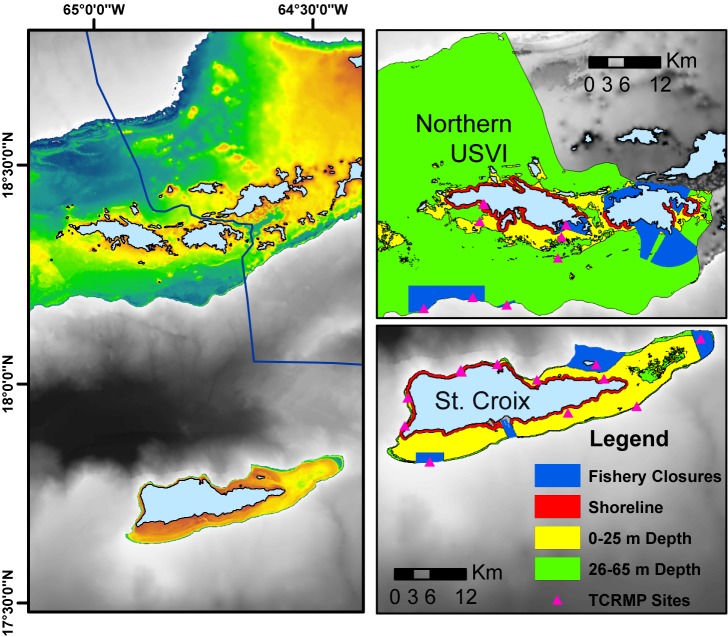
Location of northern U.S. Virgin Islands (St. Thomas and St. John) and St. Croix, separated by the Anegada passage. Right panels show the insular shelf areas of the islands separated by fishery closure areas and areas open to fishing and divided into shoreline (<500 m from shore), shallow, and deep zones. Long-term fixed sampling sites of the USVI Territorial Coral Reef Monitoring Program and included in this study are indicated.

Reef fish population declines caused by small-scale fisheries are generally fairly localized, and target species abundance can vary greatly across a region, even in areas that fall under the same fisheries laws and jurisdictions [5,12,17,18]. In such cases, the greatest resource declines are generally attributed to higher fishing pressure due to the proximity of large population centers or fishing villages [12,17,18], or the over-fishing of fish spawning aggregation sites (FSAs) to the point of fish extirpation [19–21]. The United States Virgin Islands (USVI) (Fig 1), with disparate island and submerged shelf geographies, human populations, and fishery types, provides an excellent example of how variation in these factors might influence exploitation within a small-scale reef fishery.

In the USVI, over-exploitation of commercially important reef fish species was reported as early as the late 1950s [22]. The first federal marine sanctuaries were established in 1961 off the island of St. Croix (Buck Island Reef National Monument, BINM) and 1962 off the island of St. John (St. John Biosphere Reserve, SJBS) in response to the perceived decline of fish stocks. Nevertheless, Randall reported catching substantial numbers of large grouper and snapper in shallow water off St John in the 1960s [23], suggesting that exploited fish species in nearshore shallow water were still common. At three inshore sites southwest of St. Thomas, surveyed by Rogers et al. [24] in the early 1980s, large-bodied Nassau (*Epinephelus striatus*), tiger (*Mycteroperca tigris*), and yellowfin (*M*. *venenosa*) groupers were recorded at three sites and black grouper (*M*. *bonaci*) at two sites. On St. Croix, Nassau grouper and tiger grouper were not uncommon on shallow patch reefs in Teague Bay during the same time period [25]. By the mid 1980s, however, significantly shifting fish communities were reported in both the BINM off St. Croix and the SJBR in the northern USVIs [26], with a decrease in abundance of 53% of all species in the BINM compared to surveys conducted a decade earlier [27]. Declines were particularly noted in groupers and in fish species that fed on the black spiny sea urchin *Diadema antillarum* (e.g. the queen trigger, *Balistes vetula*). In contrast, abundance of herbivores had significantly increased in both marine sanctuaries [26]. This trend has continued across the USVI [28–31]. Although a shallow water reef fisheries assessment for the US Caribbean conducted in 1991 [32] found reasonably stable catches in both the northern USVI and St. Croix between 1975 and 1989, sizes of numerous species had decreased over that time, with large snappers and groupers described as rare or commercially extinct.

Currently managed as a single unit, territorial and regional managers are now moving in a direction to separate the USVI into distinct fisheries units with regulations tailored to meet the needs and demands of the individual islands (Roy Pemberton, former Director of VI DPNR Division of Fish and Wildlife). Unfortunately, fishery-dependent data on which to make many local fishery management decisions are severely limited, and several years of data collection will be required in order to implement optimally-designed management measures. The purpose of this study is to show how population characteristics of commercially important coral reef fishes align with differences in small-scale fisheries between two areas within the USVI, the northern USVI and St. Croix, using two long-term fishery independent data sets. We hypothesized that the narrower and shallower shelf of St. Croix has allowed for more intensive fishing pressure and that this would be reflected in the abundance and sizes of large, commercially important species. We used this information to show how small-scale fisheries can differentially impact local fish populations because of likely differences in fishing intensity caused by island shelf geomorphology. Our results can serve as an example for other small-scale coral reef fisheries.

## Materials and methods

### The USVI geography, population, and fishery characteristics

The USVI population is approximately 107,000 people (USVI Census 2010) with 55,804 living in the northern USVI (St. Thomas and St. John), and 50,601 living on St. Croix. Geographically, St. Thomas and St. John lie on the eastern quarter of a large insular shelf contiguous with Puerto Rico to the west and the British Virgin Islands to the east, while St. Croix is isolated by the deep water Anegada Passage and sits on a narrow shelf 60 km to the south (Fig 1). The two island areas also have distinct shelf geomorphologies that may influence the strategies of fishers and their impact on fish populations (Table 1). The fishable area, defined as waters shallower than 65m and not in a Marine Protected Area (MPA), is approximately five times higher on the northern USVI shelf relative to the St. Croix shelf (Table 1). The shelf area by depth shows a large disparity as well, with water depth exceeding 25m making up nearly 90% of the fishable area off the northern USVI and less than 15% off St. Croix. However, the fishable area <25m depth is similar between the northern USVI and St. Croix.

**Table 1:** Submerged areas on the insular shelves of the northern USVI (N. USVI) and St. Croix with marine protected areas (MPA) and total fishable areas delineated by depth. Fishable nearshore area was defined as the coastline to 500m offshore. This is included in the 0-25m shelf, but is highlighted separately as this zone is potentially vulnerable to shore-based artisanal fisheries. Digital Elevation Model and coastline data for the US Virgin Islands and surrounding areas were obtained from the NOAA National Geophysical Data Center (www.ngdc.noaa.gov).

Fisheries District	Shelf Zone	Total Area (km^2^)	MPA Closure Areas (km^2^)	Total Fishable Area (km^2^)	Fishable Shelf by District	Fishable Shelf by USVI	MPA *
N. USVI	Nearshore	77.59	25.68 (33%)	51.91 (67%)	3.3%	2.5%	1,2
	0-25m Shelf	186.56	40.02 (21.5%)	146.54 (78.5%)	9.3%	7.1%	1,2
	25-65m Shelf	1385.23	82.69 (6%)	1302.54 (94%)	82.9%	63.5%	3
	All Shelf	1571.79	122.71 (8%)	1449.08 (92%)	92.2%	70.7%	1,2,3
St. Croix	Nearshore	54.51	2.13 (4%)	52.38 (96%)	15.1%	2.6%	4
	0-25m Shelf	297.82	37.28 (12.5%)	260.54 (87.5%)	75.3%	12.7%	4,5,6,7
	25-65m Shelf	48.22	12.27 (25%)	35.95 (75%)	10.4%	1.8%	4,5,6,7
	All Shelf	346.04	50.36 (14.5%)	296.48 (85.5%)	85.7%	14.5%	4,5,6,7
USVI Total		1917.83	172.26 (9%)	1745.57 (91%)		91.02%	

*MPAs included: 1. St. Thomas East End Reserves, 2. St. John VI National Park and Coral Reef Monument, 3. Grammanik Bank and Hind Bank, 4. Hovensa Security Zone, 5. Buck Island National Park and National Monument, 6. Lang Bank Red Hind Closed Area, 7. Mutton Snapper Spawning Aggregation

**Fig 2:**
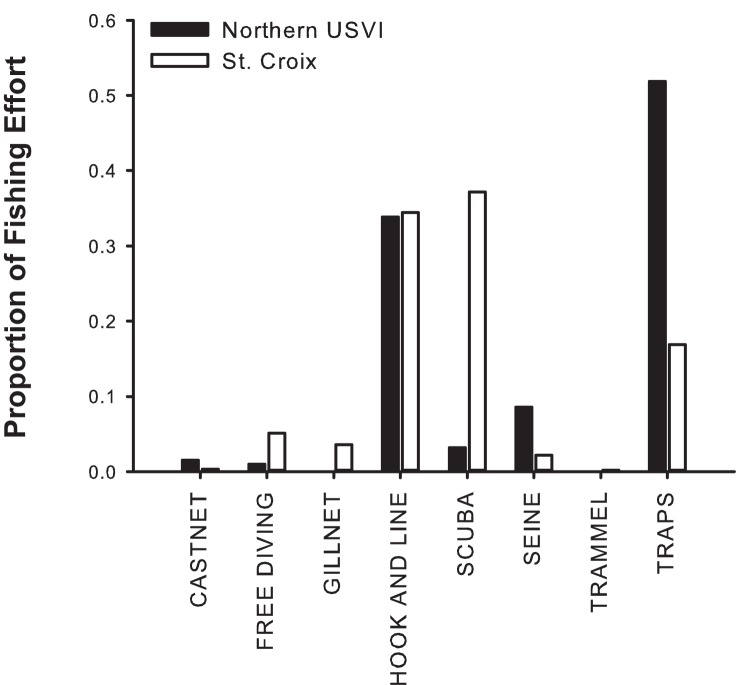
Relative fishing effort (determined by days fished) in the northern USVI and St. Croix [33].

In the USVI, reef fish management falls under the purview of the USVI Department of Planning and Natural Resources (VI DPNR) from the shoreline out to 3 miles, and the US Department of Commerce and the Caribbean Fishery Management Council (CFMC) from 3 miles to 200 miles (limit of US Exclusive Economic Zone). Currently, both the VI DPNR and CFMC manage regional waters as one management unit. Seasonal closures for grouper and snapper, size limits on yellowtail snapper and catch limits on several species are consistent across management agencies and the two island shelves. Fishing effort in the USVI, as measured by the number of registered commercial fishers, is split fairly evenly between the northern USVI and St. Croix, with approximately 90 and 121 fishers reporting catches in 2010 on each island, respectively [33]. The fisheries in the northern USVI and St. Croix can be considered distinct because fishers do not typically cross the 60 km of open water (Anegada Passage) to fish on the opposing shelf area [34]. Although fishers in both areas targets reef fish, fishing gear and practices differ markedly [34] (Fig 2). For instance, from 2001–2011 the majority of fishing trips in the northern USVI utilized fish traps, while during that same period in St. Croix, fishing effort was dominated by fishers on scuba or free diving [34]. Until very recently, accurate, reliable fisheries-dependent data were absent or sparse from the USVI [32] (Roy Pemberton, former Director of VI DPNR Division of Fish and Wildlife). Historical catch reports lumped most reef fishes into one category (potfish), and serranids (groupers) were not differentiated by species. Gaps in the database exist due to suspected inaccuracies in catch reports, and effort in the recreational fishery is completely unknown because recreational fishermen are not required to have a license or report landings. In addition, there currently is no regular port sampling program in place in the USVI, so length data for commercially exploited fishes, such has been used in the Florida Keys [35] and Puerto Rico [36] for assessment of population sustainability, are unavailable.

### Survey methods

Two separate data sets based on annual fishery-independent visual censuses over coral reefs were used to compare fish communities between the study areas. These data sets included a longitudinal data set from the USVI Territorial Coral Reef Monitoring Program (TCRMP) and a spatially stratified random data set from the US National Oceanic and Atmospheric Administration (NOAA). Differences in the TCRMP and NOAA methods lay primarily in the experimental design; sites sampled by TCRMP were fixed and sampled annually with multiple transects while NOAA sites were randomized each year using a stratified random sampling design with only one transect surveyed per site. Methods for each data set are described below.

#### Territorial Coral Reef Monitoring Program

The TCRMP began in 2003 with 14 fixed sites: 7 in the northern USVI and 7 surrounding St. Croix. The number of fixed sites expanded to 9 in the northern USVI in 2008 and 13 on St. Croix in 2009 (Table 2). Analyses presented here include data from the TCRMP database from 2003 to 2011. All sites were in scleractinian coral reef communities (>5% coral cover prior to mass bleaching and mortality in 2005) [37] and were selected as representative coral reef habitats spaced across the insular shelves of the USVI in two habitats based on water depth (<25m depth, >25 m depth). Although samples were not taken below 40m depth, the fishable area between 41–65 m depth area accounted for only about 5% of the St. Croix shelf. In the northern USVI, about 50% of the shelf area is between 41–65 m depth. However, the coral reef and hardbottom area in this depth range is only about 15% of the shelf area, since the majority of this habitat is represented by deep, flat sand plain on the north shelf and, thus, is not part of the coral reef fishery (authors, unpub. obs.). Sites were included on fish spawning aggregation areas (FSAs) in both the northern USVI (Grammanik Bank and Red Hind Marine Conservation District) and St. Croix (Lang Bank FSA and Mutton Snapper FSA), however data used here were collected outside of the spawning season for all of the grouper species.

**Table 2:** Characteristics of 22 TCRMP fixed sites used for data analysis including the island grouping used in analyses, geographic location (decimal degrees), mean depth, categorical depth used in analyses (shallow = less than 25m, deep = greater than 25m), the years surveyed in the data set, living stony coral cover prior to mass mortality of the 2005 coral bleaching event, and mean rugosity.

Site	Latitude	Longitude	Depth (m)	Depth Category	Years Surveyed	Coral Cover	Rugosity
St. Croix							
Buck Island	17.78500	-64.60917	15	Shallow	7 (2003–2005, 2008–2011)	17.3	1.42
Cane Bay	17.77388	-64.81350	10	Shallow	7 (2003–2005, 2008–2011)	23.4	1.87
Cane Bay Deep	17.77661	-64.81522	38	Deep	3 (2009–2011)	13.6	1.44
Castle	17.76278	-64.59743	7	Shallow	4 (2008–2011)	12.7	1.40
Eagle Ray	17.76150	-64.69880	10	Shallow	7 (2003–2005, 2008–2011)	7.1	1.23
Great Pond	17.71097	-64.65221	6	Shallow	6 (2003–2005, 2009–2011)	12.2	1.10
Kings Corner	17.69116	-64.90008	17	Shallow	4 (2008–2011)	17.7	1.73
Lang Bank EEMP	17.72145	-64.54706	27	Deep	3 (2009–2011)	13.3	1.18
Lang Bank FSA	17.82372	-64.44943	33	Deep	3 (2009–2011)	7.4	1.34
Mutton Snapper	17.63660	-64.86240	24	Shallow	7 (2003–2005, 2008–2011)	37.4	1.50
Salt River Deep	17.78523	-64.75917	30	Deep	4 (2009–2011)	10.1	1.74
Salt River West	17.78530	-64.75940	11	Shallow	7 (2003–2005, 2008–2011)	8.6	1.29
Sprat Hole	17.73400	-64.89540	8	Shallow	7 (2003–2005, 2008–2011)	25.6	2.38
Northern USVI							
Black Point	18.34450	-64.98595	9	Shallow	4 (2003, 2008–09, 2011)	18.4	1.39
Brewers Bay	18.34403	-64.98435	6	Shallow	4 (2003, 2008–09, 2011)	41.7	1.59
Coculus Rock	18.31257	-64.86058	7	Shallow	4 (2003, 2008–09, 2011)	11.8	1.23
College Shoal	18.18568	-65.07677	30	Deep	7 (2005–2011)	44.9	1.55
Flat Cay	18.31822	-64.99104	12	Shallow	4 (2003, 2008–09, 2011)	17.9	1.45
Grammanik Bank	18.19113	-64.95032	38	Deep	9 (2003–2011)	46.8	2.62
Hind Bank East	18.20217	-65.00158	40	Deep	9 (2003–2011)	28.0	NA
Seahorse Cottage	18.29467	-64.86750	22	Shallow	9 (2003–2011)	28.2	1.18
South Capella	18.26267	-64.87237	24	Shallow	8 (2004–2011)	35.7	1.44

Visual fish census methodology at fixed sites followed standard methods [38,39] and consisted of single divers performing 10 timed 30x 2m (2003–2008) or 10 timed 25x4m (2009–2011) belt transects to assess fish abundance and size. To perform a belt transect, a diver attached the end of a 30m transect line to the substrate at a random point and swam in a predetermined random direction, identifying fishes to the lowest taxa and counting all fish within 1 or 2m (depending on year) of either side of the transect line, including up the water column to the surface. Fish counts were placed in size bins based on total length (TL): 1-5cm, 6-10cm, 11-20cm, 21-30cm, 31-40cm, 41-50cm, 51-60cm, 61-70cm, 71-80cm and >80cm. Approximate time for each transect was 15 minutes. From 2003 to 2011, 564 and 769 transects were conducted in the northern USVI and St. Croix, respectively. In addition to transects, three replicate roving dives [40] were conducted at each site. Roving dives were 15 min (sites > 25m depth) or 30 min (sites < 25m depth) in duration. Divers swam a haphazard pattern recording all species and their overall abundance in categories, including: 1, 2–10, 11–100, 101–1000 or over 1000. Groupers, snappers, and hogfish were recorded by exact number and are presented as total counts. Overall, a total of 174 and 240 roving surveys were conducted in the northern USVI and St. Croix, respectively, between 2003 and 2011. No fish were sacrificed or handled in any way, therefore government permits were not required for the surveys. All data collected was saved and are publically available.

#### NOAA biogeography monitoring program

Data were collected by the NOAA Center for Coastal Monitoring and Assessment Biogeography Branch (NOAA CCMA) during the same time periods and in approximately the same areas. Sites were randomly selected using the random point generator in ArcView software both inside and outside of the Virgin Islands Coral Reef National Monument (VICRNM) and the BINM. Fish surveys were conducted annually from 2001–2009, generally during two consecutive weeks in July. While the design of the NOAA CCMA (hereafter, NOAA) program differed from TCRMP, visual fish census methodology was nearly identical, except that only one 15 minute 25x4m belt transect was performed on each NOAA spatially randomized site, as opposed to 10 replicates at each TCRMP fixed site. NOAA also did not sample below 30m water depth and therefore excluded much of the offshore deep habitat in both island regions. For the purpose of this study, only NOAA sites that had > 5% coral cover were used in the benthic or fish comparison since the emphasis was on coral reef-associated fisheries. The number of sites surveyed per year varied between 16 and 59 in St. Croix and 17 and 61 in the northern USVI. This amounted to a total number of sites surveyed in St. Croix and the northern USVI of 348 and 357, respectively.

#### Benthic composition between methodologies and island shelves

To test for the influence of habitat on fish community structure apart from fishing, we measured total coral cover at TCRMP fixed sites using video sampling along 10m transects following standard methodologies as described in Smith et al. [41]. Total coral cover was visually assessed on NOAA spatially randomized sites by placing a 1m^2^ quadrat at five separate, randomly located positions along a 25m belt transect [42]. Coral cover was compared between fixed sites and spatially-randomized sites and between the northern USVI and St. Croix using a two-way ANOVA.

Rugosity was also measured at TCRMP fixed sites in 2011 with three 3m chain transects laid along permanent transects and expressed as the ratio of the contour following chain to straight distance along the transect, with the exception of the northern USIVI site Hind Bank East (Table 2). The missing Hind Bank East site rugosity value was filled in with the value of a similar orbicellid mesophotic reef, Grammanik Bank. This was considered conservative for this comparison since the Grammanik Bank had the highest rugosity value in the dataset and would exaggerate the value of the northern USVI mean rugosity, hence the difference in structure available for fishes.

#### Target species

Fourteen reef fish species were selected for analysis due to their presence in the data, commercial importance in the USVI fisheries, and/or their ecological importance as top or intermediate predators in Caribbean coral reef fish communities. These species included cubera snapper (*Lutjanus cyanopterus*), dog snapper (*L*. *jocu*), gray snapper (*L*. *griseus*), lane snapper (*L*. *synagris*), mutton snapper (*L*. *analis*), schoolmaster snapper (*L*. *apodus*), yellowtail snapper (*Ocyurus chrysurus*), Nassau grouper, tiger grouper, yellowfin grouper, yellowmouth grouper (*M*. *interstitialis*), red hind (*E*. *guttatus*), coney (*Cephalopholis fulva*), and hogfish (*Lachnolaimus maximus*). These fishes were all found in very low densities across both the fixed and spatially-randomized sites, and therefore a relative encounter metric was used in place of density in analyses. On TCRMP fixed sites, this metric was calculated as the % of all transects in which that species was encountered per site per year. Because only one transect was sampled per site in the NOAA spatially-randomized dataset, the relative encounter metric was calculated as the % of all sites (equivalent to % of all transects) in which that species was encountered. This resulted in only one relative encounter value per species per year, and so data were not analyzed for temporal trends, but graphical inspection revealed no large differences in encounter rates through time.

#### Fish abundance data analysis

In order to test the hypothesis that St. Croix has relatively less abundant target species than the northern USVI, we compared relative encounter data at TCRMP and NOAA sites separately. Relative encounters for each species did not conform to assumptions of parametric tests, therefore, non-parametric techniques were applied. For NOAA data, all were taken shallower than 30m depth and this was treated as shallow habitat and non-parametric Wilcoxon tests were used to compare islands (St. Croix versus northern USVI). For the TCRMP data, Friedman’s rank tests were first tested to examine for any effects of time, but no significant trends in time were found for all fourteen species (p > 0.05 for all tests). Therefore, time was not treated as a separate effect in further analyses of these fixed site data. However, the TCRMP data were collected at depths from 6-40m, which allowed for an analysis of the effect of depth on the relative encounters of species of interest. The depth treatment was divided into the levels shallow (<25 m) and deep (>25m) and used to categorize sites (Table 2) resulting in 3 shallow and 6 deep sites in the northern USVI and 4 deep and 9 shallow sites in St. Croix. TCRMP relative encounter data by site was then analyzed as a two-way Friedman’s Rank test on ranked data with treatments: island, depth, and the interaction of island and depth. Significant interaction terms were analyzed with a Tukeys Post Hoc HSD Test. Statistical analyses of species relative encounters were performed using the software package JMP v. 10 or 13.0.0 (SAS Institute Inc.). In addition to relative encounter analysis, the total number of large groupers, snappers and hogfish observed on TCRMP roving dives were tabulated by site and year for each of the two study areas and graphed to show obvious trends. Rare species included Nassau grouper, tiger grouper, yellowfin grouper, yellowmouth grouper, cubera snapper, dog snapper, mutton snapper, and hogfish. These were graphed to illustrate differences in the occurrence of these species across island shelves.

#### Multivariate data analysis

Relative encounters (as defined above under “Target Species”) for all fourteen fish species were analyzed simultaneously as an assemblage for differences between islands using non-metric multidimensional scaling (MDS) ordinations based on a Bray-Curtis similarity matrix. Fixed site TCRMP and spatially-randomized NOAA datasets were analyzed separately. In the fixed site dataset, relative encounter assemblages did not change through time for the northern USVI (R = 0.057, p = 0.11) or St. Croix (R = 0.075, p = 0.22); therefore, relative encounters were averaged across years to be used in the analysis for differences between islands. Differences in relative encounter assemblages between islands were statistically compared using the Analysis of Similarities (ANOSIM) technique. ANOSIM is analogous to single response ANOVA but applied to multivariate datasets, particularly with respect to community data [43]. When a significant ANOSIM was detected, it was followed by a similarities percentages (SIMPER) technique to determine which species contributed the most to statistical differences [43]. We did not directly compare relative encounters between TCRMP fixed site and NOAA spatially-stratified datasets, but used the two datasets as a qualitative comparison to test the generality of our findings. Multivariate analyses were performed using PRIMER 6 (v. 6.1.13).

#### Length frequency data analysis

In order to test the hypothesis that sizes of target fish species were smaller (left-skewed) on St. Croix relative to the northern USVI, we statistically compared the size-structure distribution of each species and then examined the plots. Four species of fish with abundances within the data set that were sufficiently high to allow reliable statistical testing were used to compare length frequency distributions measured as the percentage of the population distributed among size classes between island groups. These species included red hind, yellowtail snapper, coney and schoolmaster. These species were the only members of the target group represented in the TCRMP fixed site belt transect database with more than 100 individuals per species. Data sets were combined across the two study areas and across all years. Length frequency distributions of these four species were also compared between islands in the spatially-randomized database, with all years combined. Statistical comparisons of frequency distributions were made using Kolmogorov-Smirnov tests in JMP v. 10 (SAS Institute Inc.). Where statistical differences were found, the plots of size distribution were inspected to look for evidence of increasing right- or left-skewness.

## Results

**Fig 3:**
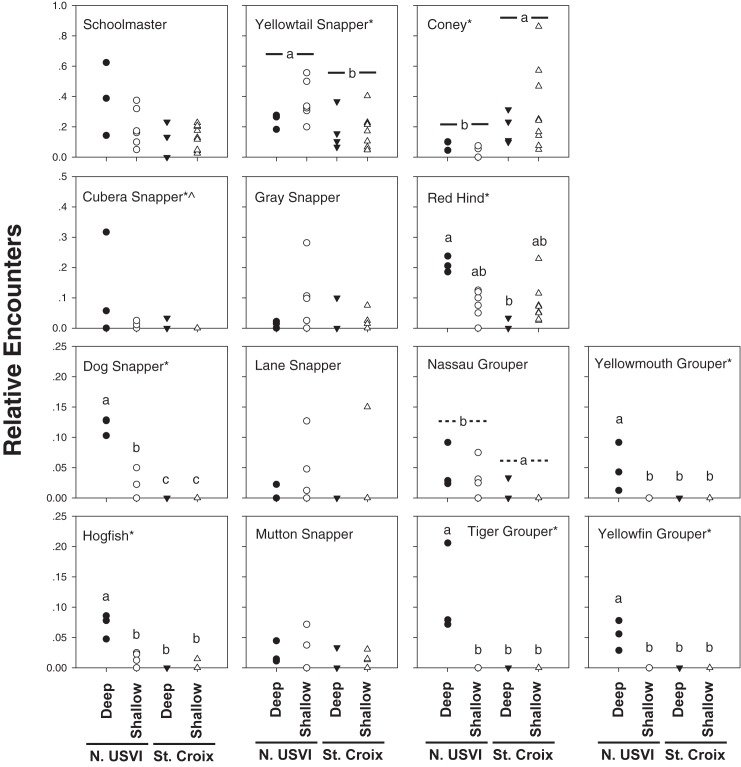
Relative encounters (% of transects in which the fish species was seen) for the fourteen target species at fixed sites of the USVI Territorial Coral Reef Monitoring Program. Comparisons were made between island group and depth (<25 m and >25m). Asterisks (*) following species names indicate a significant overall model (α = 0.05). Carat symbol (^) for cubera snapper indicates that both depth and island were significant. Post-hoc analyses indicated as disparate letters over treatments where there was interaction between island and depth, and as a joining line where there was a significant across island effect. Nassau grouper was marginally non-significant for the overall model (p = 0.0567), but post-hoc analyses are presented for island and indicated with a dashed joining line.

**Fig 4:**
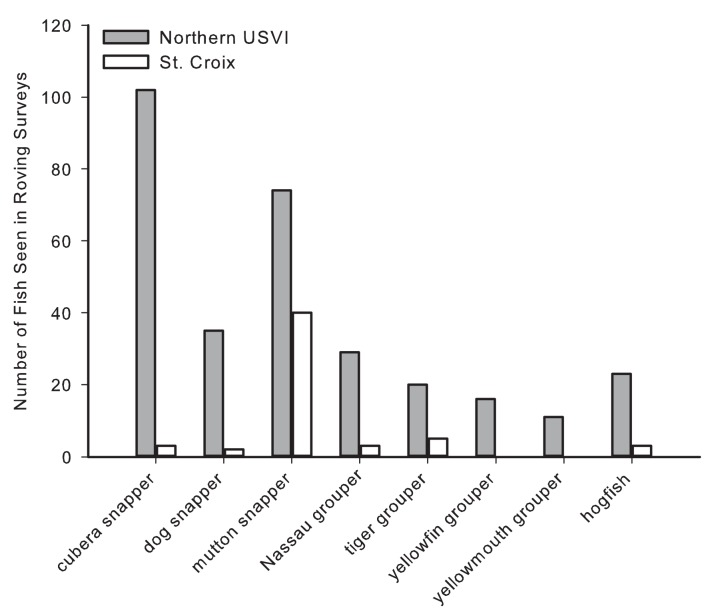
Total number of fish encountered in roving dives for eight of the large-bodied commercially important species in the northern USVI and St. Croix. Data from fixed sites of the USVI Territorial Coral Reef Monitoring Program conducted between 2003–2011 (N_N USVI_ = 174, N_St. Croix_ = 240).

Large-bodied commercially important snappers, groupers, and hogfish were greatly reduced or absent in St. Croix compared with the northern USVI (Fig 3). In six of fourteen species, there was an interaction of island and depth (Table 3), with post-hoc analyses indicating that the deep northern USVI had higher relative encounters for tiger grouper, yellowfin grouper, yellowmouth grouper, dog snapper, hogfish, and red hind (Fig 3). Yellowtail snapper was more abundant in the northern USVI in general, whereas cubera snapper were more abundant in the northern USVI, and separately in deep zones in the northern USVI and St. Croix. There was a similar pattern of higher abundance of Nassau grouper in the northern USVI, but the full model was marginally not significant (p = 0.0567). Coney was the only fish species more abundant in St. Croix than the northern USVI, regardless of depth. Results of relative encounters in TCRMP data were supported for eight rare species in roving dives (Table 4, Fig 4). There were only two individuals of large groupers encountered on roving dives in St. Croix, whereas in the northern USVI there were a total of 67 encounters, including 28 Nassau grouper, 17 tiger grouper, 13 yellowfin grouper, and 9 yellowmouth grouper. The result for Nassau grouper in particular indicates that this species is more abundant in the northern USVI, even though TCRMP relative encounter data were marginally non-significant. Cubera snapper, dog snapper, and hogfish all showed a pattern that supported the TCRMP relative encounter data, with the exception of a higher number of dog snapper and hogfish encountered on northern USVI roving dives in shallow water. As also seen in TCRMP relative encounters, mutton snapper were equally abundant in both St. Croix and the northern USVI roving dives.

**Table 3:** Results of statistical analysis of relative encounters of fourteen large and medium-bodied commercially important species on belt transects at fixed sites of the USVI Territorial Coral Reef Monitoring Program. Comparisons were two-way Friedmans Rank test comparing the treatments Island (St. Croix and the northern USVI), Depth (less than 25m and greater than 25 m), and their interaction (Island*Depth). All degrees of freedom were 3/18 (numerator/denominator) for the whole model and 2 for individual treatments. Where the model was not statistically significant the individual tests of treatments are not presented. Bold indicates significant treatments or interactions that were followed up with pair-wise tests.

Common Name	Treatment	F/t*	p-value
Hogfish	Model	23.98	<0.0001
	Island	50.6	<0.0001
	Depth	19.8	0.0003
	Island*Depth	23.9	**<0.0001**
Coney	Model	8.26	0.0011
	Island	4.03	**0.0008**
	Depth	0.75	0.4603
	Island*Depth	1.28	0.2180
Nassau Grouper	Model	3.02	0.0567
	Island	5.87	**0.0261**
	Depth	2.56	0.1269
	Island*Depth	0.05	0.8217
Red Hind	Model	4.32	0.0184
	Island	10.80	0.0041
	Depth	0.50	0.4879
	Island*Depth	6.23	**0.0225**
Tiger Grouper	Model	31.09	<0.0001
	Island	41.81	<0.0001
	Depth	41.81	<0.0001
	Island*Depth	41.81	**<0.0001**
Yellowfin Grouper	Model	31.09	<0.0001
	Island	41.81	<0.0001
	Depth	41.81	<0.0001
	Island*Depth	41.81	**<0.0001**
Yellowmouth Grouper	Model	31.09	<0.0001
	Island	41.81	<0.0001
	Depth	41.81	<0.0001
	Island*Depth	41.81	**<0.0001**
Cubera Snapper	Model	5.08	0.0101
	Island	6.52	**0.0200**
	Depth	9.10	**0.0074**
	Island*Depth	2.64	0.1216
Dog Snapper	Model	24.30	<0.0001
	Island	61.06	<0.0001
	Depth	13.45	0.0018
	Island*Depth	13.45	**0.0018**
Gray Snapper	Model	2.50	0.0927
Lane Snapper	Model	0.68	0.5763
Mutton Snapper	Model	0.48	0.6997
Schoolmaster Snapper	Model	1.53	0.2412
Yellowtail Snapper	Model	3.43	0.0392
	Island	6.09	**0.0238**
	Depth	0.75	0.3960
	Island*Depth	0.89	0.3571

**Table 4:** Number of encounters of eight rare species in both roving dives and belt transects conducted by TCRMP from 2003–2011. Encounters are reported by study area (Northern USVI and St. Croix) and strata. Years are collapsed. X indicates no encounters.

Method	Species	Shallow	Deep	Shallow	Deep
**Roving Dives**		**N = 99**	**N = 75**	**N = 192**	**N = 48**
	Nassau grouper	9	17	X	1
	Yellowfin grouper	1	11	X	X
	Tiger grouper	1	16	X	X
	Yellowmouth grouper	1	7	X	1
	Cubera snapper	3	21	6	2
	Mutton snapper	42	16	21	9
	Dog snapper	20	32	1	3
	Hogfish	19	10	2	X
**Belt Transects**		**N = 223**	**N = 241**	**N = 614**	**N = 155**
	Nassau grouper	5	12	X	1
	Yellowfin grouper	X	14	X	X
	Tiger grouper	X	30	X	X
	Yellowmouth grouper	X	2	X	X
	Cubera snapper	3	33	X	2
	Mutton snapper	9	6	5	2
	Dog snapper	10	29	X	X
	Hogfish	4	17	1	X

**Fig 5:**
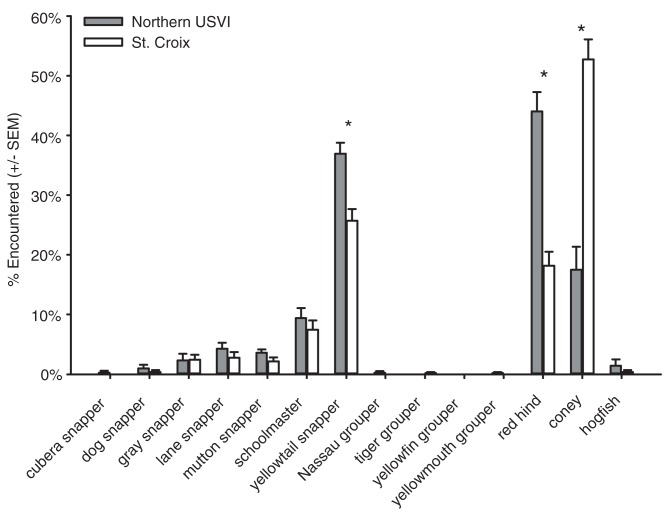
Relative encounters for the fifteen target species in NOAA spatially-randomized transects conducted in shallow water less than 30 m depth (N = 10 for both the northern USVI and St. Croix). * indicates a significant difference (p < 0.05) between Northern USVI and St. Croix relative encounters for that species as determined by a Wilcoxon test (statistical results in Table 5).

Spatially randomized surveys conducted by NOAA corroborated the shallow water results from the TCRMP data. In the NOAA spatially-randomized surveys of coral reefs in water depth less than 30m, 23 large-bodied grouper species were recorded in the northern USVI, and only 4 were encountered off St. Croix. Relative encounters of the fourteen large- and intermediate-bodied focal species on shallow NOAA belt transects showed that red hind and yellowtail snapper were significantly more abundant in the northern USVI, whereas coney were significantly more abundant in St. Croix (Fig 5, Table 5).

**Table 5:** Results of statistical analysis of relative encounters of fourteen large and medium-bodied commercially important species in NOAA spatially-randomized transects conducted in shallow water less than 30 m depth (N = 10 for both the northern USVI and St. Croix). Comparisons were Wilcoxon rank tests comparing island (St. Croix and the northern USVI). Bold indicates significant differences (p < 0.05).

Common Name	Z	P
Hogfish	-0.669	0.5036
Coney	3.666	**0.0002**
Nassau Grouper	-0.900	0.3681
Red Hind	-3.666	**0.0002**
Tiger Grouper	0.900	0.3681
Yellowfin Grouper	N/A	N/A
Yellowmouth Grouper	-0.900	0.3681
Cubera Snapper	-0.900	0.3681
Dog Snapper	-0.669	0.5036
Gray Snapper	0.444	0.6569
Lane Snapper	-0.958	0.3383
Mutton Snapper	-1.601	0.1094
Schoolmaster Snapper	-0.757	0.4494
Yellowtail Snapper	-3.064	**0.0022**

**Fig 6:**
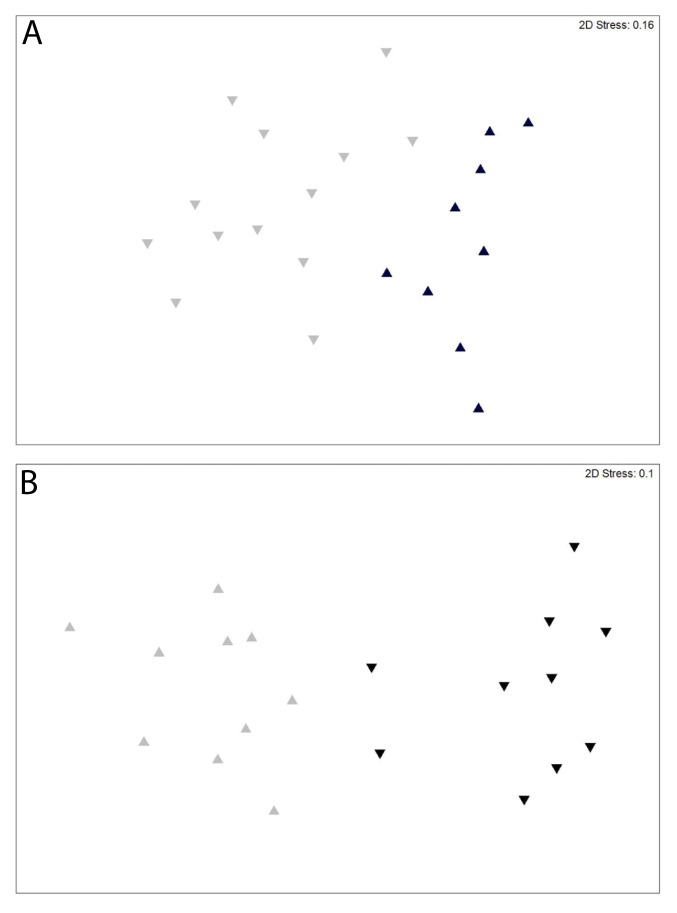
MDS ordinations of target species assemblages between northern USVI and St. Croix based on (A) fixed site data and (B) spatially-randomized data. Black triangles indicate northern USVI sites; Gray triangles indicate St. Croix sites. The 3D plots did not improve stress greatly (3D Stress = 0.11 and 0.06, respectively) and therefore are not presented.

The MDS ordinations of fish assemblages for both the fixed site and spatially-randomized relative encounter datasets showed a clear differentiation between the northern USVI and St. Croix sites (Fig 6). These differences were both statistically significant (Fixed site ANOSIM: R = 0.557, p < 0.001; Spatially-randomized ANOSIM: R = 0.862, p < 0.001). The average dissimilarity calculated between islands was 58.8% and 39.8% for the fixed site and spatially-randomized datasets, respectively. In both datasets, coney and yellowtail snapper were in the top three species that contributed the most to dissimilarity between the northern USVI and St. Croix (Table 6). Schoolmaster snapper and red hind were the third species that contributed the most to dissimilarity between northern USVI and St. Croix in the fixed site and spatially-randomized datasets, respectively (Table 6).

**Table 6:** Results of the SIMPER analysis between islands based on fixed (TCRMP) and spatially randomized (NOAA) datasets. Fish species listed are the top five species that contributed the most to dissimilarity between the northern USVI and St. Croix multivariate data sets of species relative encounters.

Method	Species	Northern USVI Average % Encounter	St Croix Average % Encounter	Average Dissimilarity	Ave Diss Standard Variation	% Contribution	Cumulative % Contribution
TCRMP	Coney	0.04	0.28	13.10	1.24	22.29	22.29
	Yellowtail snapper	0.33	0.17	12.51	1.20	21.29	43.58
	Schoolmaster snapper	0.26	0.13	9.36	1.31	15.92	59.50
	Red hind	0.12	0.06	5.07	1.59	8.62	68.12
	Gray snapper	0.06	0.02	3.62	0.86	6.16	74.28
NOAA	Coney	0.53	0.17	15.24	2.23	38.25	38.25
	Red hind	0.18	0.44	11.09	2.29	27.84	66.08
	Yellowtail snapper	0.26	0.37	5.17	1.61	12.97	79.05
	Schoolmaster snapper	0.07	0.09	2.48	1.48	6.23	85.29
	Lane snapper	0.03	0.04	1.56	1.26	3.91	89.19

**Fig 7:**
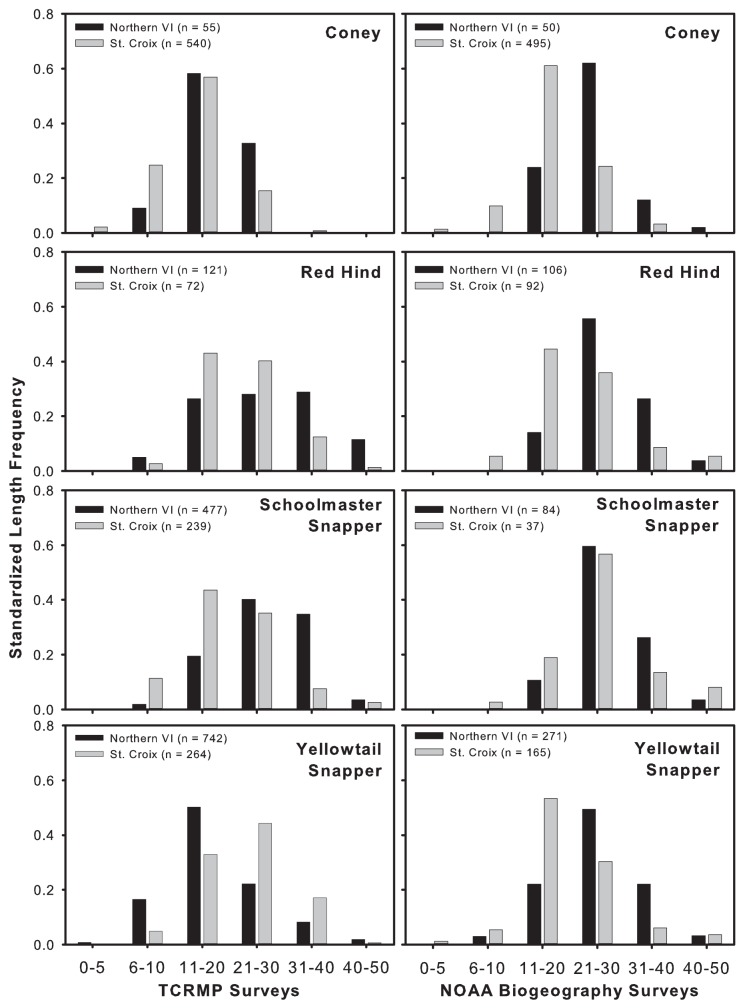
Total length (cm) frequency distributions for four common commercially important species based on fixed site (left column) and spatially-randomized (right column) surveys between the northern USVI (dark bars) and St. Croix (gray bars). N indicated in graphs are number of fish.

Length frequencies of three fish species of the four compared tended to show that larger fishes were more common in the northern USVI compared to St. Croix (Fig 7). Length frequency distributions differed significantly between the northern USVI and St. Croix for coney, red hind, and yellowtail snapper in the fixed site database and for coney and yellowtail snapper in the spatially-randomized data set (Table 7). Coney and red hind were larger in the northern USVI, as seen as a higher frequency of individuals in the largest size classes in both data sets. Yellowtail snapper showed opposing trends depending on method, with the fixed site data showing larger fish on St. Croix and the spatially randomized data set showing larger fish in the northern USVI. However, this trend was less apparent in the largest size class (40-50cm). The frequency distribution of schoolmaster snapper lengths was not significantly different between islands (Fig 6).

**Table 7:** Results of Kolmogorov-Smirnov tests using fixed site (TCRMP) and spatially-randomized (NOAA) data sets comparing size frequency distributions between the northern USVI and St. Croix.

Species	Method	KS	p
Yellowtail Snapper	Fixed site	1.60	< 0.01*
	Spatially-randomized	1.34	< 0.05*
Red Hind	Fixed site	1.34	< 0.05*
	Spatially-randomized	0.53	0.056
Schoolmaster	Fixed site	0.80	0.54
	Spatially-randomized	1.07	0.20
Coney	Fixed site	1.34	< 0.05*
	Spatially-randomized	1.34	< 0.05*

* Indicates where a significant (p < 0.05) difference in size distributions occurred between the two study areas.

In the analysis of habitat factors, and in testing for coral cover differences between data sets and island areas, the overall two-way ANOVA was significant (F = 7.79, d.f. = 3/545, p < 0.001), and identified that coral cover was higher at TCRMP fixed sites than at NOAA spatially-randomized sites (t = -4.49, p < 0.001), but coral cover did not vary by island (t = 0.26, p = 0.79) and there was no interaction of island and method (t = -0.82, p = 0.41). Comparison of islands also showed no significant difference in overall rugosity as measured at TCRMP fixed sites (Rugosity_St. Croix_ = 1.51 ± 0.10 SE, Rugosity_N. USVI_ = 1.67 ± 0.18; t-test_two-tailed_ = 0.78, p = 0.450).

## Discussion

This study reaffirms the over-exploitation of commercially important species in the USVI, which appears to have been especially detrimental to populations of large predatory fishes.Although habitat-related variables such as coral cover, rugosity, habitat complexity, and seascape patterns are important factors shaping the reef fish communities, these variables have not changed markedly since the 1970s, in contrast to the fisheries [32, 44]. Large-bodied Nassau, yellowfin and tiger groupers that were once fairly common in shallow, nearshore coral reefs across the territory, were nearly absent in the study except on deepwater reefs on the outer shelf of the northern USVI. With the exception of mutton snapper, the encounter rate of most large-bodied snapper and grouper on the St. Croix shelf was nearly zero or zero. It is more difficult to ascertain whether the intermediate-sized commercially important species are reduced compared to historical abundance. However, there was evidence that lane snapper, schoolmaster, yellowtail snapper and red hind, were all lower in abundance on St. Croix relative to the northern USVI, and that red hind and coney had lower representation in the largest size classes on the St. Croix shelf relative to the northern USVI.

We suggest that fishing intensity is driving differences in abundance of commercially important fish species between the two island shelves. The disparity between fish communities and their relative changes over recent decades can not be explained by local territorial fisheries regulations, which are consistent across the territory. In addition, MPAs cover more relative area in St. Croix than the northern USVI. Habitat can be a potent driver of fish communities in coral reefs, with habitat complexity, not coral cover *per se*, indicated as highly important [45, 46]. In our study however, mean site coral cover and mean rugosity was not different between coral reefs monitored on the island shelves, indicating similar habitat complexity in the northern USVI and St. Croix. We suggest instead, that differences in the occurrence of top and intermediate predators between the island shelves may be driven by a combination of several other factors that change fishing pressure, including water depth, fishing methodology, fishable area and the presence or absence of viable FSAs.

Large differences in the overall area and water depth of the insular shelves may stimulate the use of different fishing methods and cause increased relative fishing pressure on St. Croix that is driving greater exploitation of fishes. The similarity in the number of registered fishers between the northern USVI and St. Croix suggests that differences in fishing intensity are not due to numbers of fishers, although numbers of fishers is only a proxy for fishing intensity. St. Croix has approximately the same nearshore and shallow water (<25 m depth) fishable shelf area as the northern USVI. However, the northern USVI has 37 times more fishable deep shelf between 25 to 65 m than St. Croix and a total fishable area that is almost 5 times greater. Not only does this mean more area per fisher, but it also provides a much larger deep water shelf area that is more difficult to fish with active, labor intensive methods, such as scuba and hook and line. The predominance of shallow shelf off St. Croix that allows for a commercial fishery based on spearfishing and collecting while using scuba is limited on the northern USVI due to greater water depths. In addition, because there still remain high catches with other gears (e.g., Antillean fish traps), it might be unnecessary for fishers to engage in more intensive and risky scuba fishing in the shallow shelf areas of the northern USVI where it might be viable. In the past several years, the only fatalities or serious injuries in the USVI commercial fishery were spearfishers, and the vast majority of them on were on St. Croix using scuba gear [47]. The level of exploitation caused by commercial and recreational spearfishing compared to other fishing methods is controversial, but there is general agreement regarding the increased time efficiency and selectivity of the gear, especially when harvesting large commercially valuable species such as groupers and snappers, and when fishing productive sites (FSAs and fish movement/migration pathways) [48–52]. Unlike other fisheries that generally switch target species as catch rates become low, commercial spearfishing with scuba continues to be economically viable even after fish abundances are well below the level needed for successful reproduction [50]. Perhaps in recognition of spearfishing’s impact on fish populations, in much of the Indian Ocean and Caribbean where tourism plays an important role in the economy, island nations have banned or highly regulate spearfishing activities (i.e. Maldives, Bahamas, Cayman Islands, Netherland Antilles, British Virgin Islands, Antigua/Barbuda). Where spearfishing is the primary gear used in local small-scale fisheries, such as many Pacific nations, the use of scuba and hookah assistance is banned [52]. This includes the nations of Fuji, Samoa, Palau, Tonga, the Solomon Islands and American Samoa. Much like the shallow atolls of the Pacific, the St. Croix shelf may be particularly vulnerable to scuba-assisted spearfishing, with a paucity of expansive deepwater mesophotic reefs like those found in the northern USVI [37]. This reduces the potential for a depth refuge for reef fishes [51, 53] and subjects them to the constant pressure of shallow water activities such as scuba-assisted spearfishing.

In contrast, two marine protected areas in the northern USVI encompass deep grouper and snapper spawning aggregation sites, and large tracts of seafloor that surround these sites. The establishment of the Grammanik Bank in 2005 may have played a role in the increased number of groupers observed on the northern USVI shelf. The grouper species Nassau, yellowfin, and tiger and the snapper species dog and cubera use the Grammanik Bank for spawning [54] (although observations for this study occurred outside of spawning periods). The large (49 km^2^), offshore, deep, Red Hind Marine Conservation District no-take reserve located less than 5km to the west of the Grammanik Bank may also provide safe corridor for fish from the Virgin Passage arriving and departing the Grammanik Bank spawning site [55]. This reserve may also provide large protected home range areas for grouper and snapper. No large-bodied grouper (e.g., Nassau, yellowfin, tiger) spawning is currently known to occur off St. Croix (an anecdotal Nassau grouper FSA reportedly located on the Lang Bank was fished out in the 1970s). Likewise, spawning aggregations of all of the large-bodied snappers (cubera, dog and mutton) have been observed and documented in the northern USVI [56], however functioning snapper FSAs are unknown for all but the mutton snapper off St. Croix. This may reflect targeting of aggregations by fishers and general overexploitation that pushed fish populations below a critical density for aggregation formation.

FSA loss can also have secondary effects by increasing recruitment failure. Due to planktonic larvae and significant ocean currents, local fish assemblages in the Caribbean may reflect to some extent processes operating hundreds of kilometers upcurrent or on distant neighboring platforms and reefs [57, 58]. However, local larval retention is believed to be a key driver for population persistence [59–62] especially in isolated, insular ecosystems. As such, self seeding and recruitment of groupers and snappers to the coral reef communities around St. Croix may be highly compromised with the absence of local viable aggregations. The exception on St. Croix is a mutton snapper FSA which is protected seasonally [63]. Unlike all other grouper and large snapper species, encounters with mutton snapper were not significantly different between islands in either TCRMP fixed site or NOAA randomly stratified databases, suggesting FSA protection is working for this species.

The significantly greater abundance of coney, an intermediate predator, on St. Croix may be the result of overfishing the larger groupers and snappers. Previous studies have noted that ecological extinctions of large-bodied predators may result in intermediate sized predators being released from predation or competition (i.e. mesopredator release), and thus increasing in abundance [63]. For example, Sluka et al. [64] and Chiappone et al. [65] reported inversely correlated abundances of coney and Nassau grouper in the Bahamas and Florida Keys, and Stallings [66] documented both a change in behavior (more time hiding) and decrease in individual fish biomass of coney with the addition of Nassau grouper to coral reefs in the Bahamas. This in turn positively influenced recruitment of several other species of reef fish. This shift of community structure with the loss of top down control and its implications on coral reefs is complex and very poorly understood. However, over-exploitation and trophic shifts in fish and invertebrate community composition can in turn change biological processes that effect primary productivity, coral cover and health [67, 68], and eventually fundamental physical processes such as calcium carbonate accretion and bioerosion [69]. Although logistically challenging, increased research is necessary to improve our understanding of the more pervasive effects of predator removal on community dynamics, and the consequential ecological and economic compromises imposed on coral reefs.

Region-wide declines in reef fish have now been documented across the wider Caribbean, with declines attributed to habitat-related changes [6], high population densities [70] and overfishing [8]. Hawkins and Roberts [8] found order of magnitude declines in biomass of fisheries species and significant declines in fish abundance and species richness as fishing intensity increased across a range of six separate Caribbean Islands. Large groupers had been extirpated from all but the two most lightly fished areas, Bonaire and Saba. In addition, declines in biomass of several families (groupers, snappers, parrotfishes and doctorfishes) were differential across species, depending on size, with the largest species in the family declining the fastest as fishing pressure increased. We believe that St. Croix may be an example of a severely overfished Caribbean island, and this study illustrates the need for management of artisanal fisheries that is tailored to the physical and spatial constraints imposed by shallow insular platforms. The small isolated shelf surrounding St. Croix in particular represents a very complex management challenge that must balance no-take conservation areas with decreasing fishable seafloor and a concentration of remaining fishing effort. Along with well-designed and strictly enforced no take areas that include viable FSAs, fishing pressure needs to be addressed. The use of scuba for commercial spearfishing should be closely examined for its potential role in the overexploitation of large-bodied snappers and groupers. Furthermore, a ban on the harvest of all large-bodied grouper species may be necessary to help to reestablish populations and self-sustaining spawning aggregations.
